# Application of Impermeable Barriers Combined with Candidate Factor Soaked Beads to Study Inductive Signals in the Chick

**DOI:** 10.3791/54618

**Published:** 2016-11-17

**Authors:** Susan Wilde, Malcolm P. Logan

**Affiliations:** ^1^Randall Division of Cell and Molecular Biophysics, King's College London, Guy's Campus

**Keywords:** Developmental Biology, Issue 117, Chick embryo, Impermeable barriers, Gene expression, Retinoic acid signaling, Somites, Lateral plate mesoderm, Limb induction, Limb initiation, Tbx5, Tbx4

## Abstract

The chick embryo provides a superb vertebrate model that can be used to dissect developmental questions in a direct way. Its accessibility and robustness following surgical intervention are key experimental strengths. Mica plates were the first barriers used to prevent chick limb bud initiation^1^. Protocols that use aluminum foil as an impermeable barrier to wing bud or leg bud induction and or initiation are described. We combine this technique with bead placement lateral to the barrier to exogenously supply candidate endogenous factors that have been blocked by the barrier. The results are analyzed using *in situ* hybridization of subsequent gene expression. Our main focus is on the role of retinoic acid signaling in the induction and later initiation of the chick embryo fore and hindlimb. We use BMS 493 (an inverse agonist of retinoic acid receptors (RAR)) soaked beads implanted in the lateral plate mesoderm (LPM) to mimic the effect of a barrier placed between the somites (a source of retinoic acid (RA)) and the LPM from which limb buds grow. Modified versions of these protocols could also be used to address other questions on the origin and timing of inductive cues. Provided the region of the chick embryo is accessible at the relevant developmental stage, a barrier could be placed between the two tissues and consequent changes in development studied. Examples may be found in the developing brain, axis extension and in organ development, such as liver or kidney induction.

**Figure:**
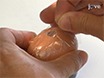


## Introduction

Classical embryologists traditionally employed physical techniques to interrogate the mechanisms controlling embryonic development. In the 1960s and 1970s investigators developed techniques that used impermeable barriers inserted between tissues of the developing embryo to demonstrate the importance of inductive signals during embryogenesis. Our interest is in vertebrate limb development and in particular what events precede limb bud outgrowth. For example, insertion of a barrier can prevent limb bud outgrowth from occurring on one side of the chick embryo while allowing it to proceed normally on the contralateral, unoperated side. Materials used to make these barriers varied; for example, mica plates^ 1^ and tantalum foil ^2^. These barriers effectively separate the lateral plate mesoderm (LPM), from which the limb bud forms, from the somites formed from the paraxial mesoderm. Surgical grafting techniques demonstrate that close association with the somites is essential if limb bud initiation is to occur in the LPM ^3^^4^. In the 80s and 90s developmental biologists discovered diffusible signaling molecules that are essential in controlling developmental processes. Beads soaked with these signaling molecules can be implanted in specific regions of the embryo and produce alterations to embryonic development. For example, retinoic acid (RA) taken up by ion exchange beads is released over a 24-hour period when implanted into a chick embryo and can produce mirror-image duplication of the digits ^5^. Heparin acrylic beads containing FGF protein can initiate limb bud outgrowth from the interlimb LPM ^6^.

More recently mouse and fish genetics have taken the study of vertebrate limb initiation into a new era (reviewed in ^7^). There is good evidence that RA present in the somites prior to limb bud initiation is the essential diffusible signal required by the LPM to initiate limb budding. We wanted to exploit the accessibility and experimental robustness of the chick embryo to dissect the effect of barrier implantation on gene expression in the LPM around the time of limb bud initiation. A novel adaptation of our method is to combine a barrier that blocks signals from axial tissues with bead placement lateral to the barrier to exogenously supply candidate endogenous factors that have been blocked by the barrier. The following protocols are those developed to tease out the mechanisms of limb bud induction and initiation.

Studying limb bud initiation means using early stage embryos. Many researchers window chicken eggs by first removing some of the albumin through the air sac with a hypodermic needle. The embryo, which lies on top of the yolk, will then lie lower in the egg. This is an advantage for some techniques, such as electroporation, but can be a disadvantage if surgical manipulation of the embryo is desired. We find having the embryo as close to the opening in the egg as possible is an advantage.

Questions remain regarding the signals that control vertebrate limb bud induction and initiation and the following protocols are suitable to address them. We are the first to develop a protocol for the insertion of barriers to prevent chick limb bud outgrowth at a stage earlier than stage 12 ^8^. Modified versions of these protocols could also be used to address other questions on the origin and timing of inductive cues where an accessible inducing tissue lies adjacent to the primordium being induced. Provided the region of the embryo is accessible at the relevant developmental stage, then a barrier could be placed between the two tissues and consequent changes in development studied. Examples may be found in brain development, axis extension and possibly organ development such as liver or kidney induction.

## Protocol

The following protocol uses chicken embryos at less than ten days of incubation. All experiments were performed in accordance with King's College London, UK and the UK Home Office animal care guidelines.

### 1. Preparation Required Prior to Chick Embryo Operation

1. Gather the following surgical instruments required for the operations, for harvesting and for fixing the chick embryos: Two pairs of No. 5 watchmakers forceps, one pair of strong forceps, *e.g.*, type AA, curved scissors with blades 2.5 cm long, blunt ended curved forceps with ends approximately 0.5 cm long, and also two pairs of No. 4 watchmakers forceps to use while making foil barriers (1.6.3).
2. Make a micro knife from a steel pin (0.3 mm across) sharpened using oil on a sharpening stone. Using a binocular microscope, sharpen the tip of the pin to a flat blade. Place the pin in a needle holder and protect its sharp end from being knocked and dented. NOTE: Sheathing the knife with a 1,000 µl pipette tip is a simple option for this.
3. Prepare 70% ethanol in a plastic wash bottle. Use a squirt of this on a paper tissue to wipe the surgical instruments and the micro knife before and after use to sterilize and clean them.
4. Buy 5 cm wide clear sticky tape and a suitable dispenser. Find or manufacture a rest suitable to hold a chicken egg steady on its side.
5. Make and store solutions for soaking beads.
  1. Prepare a 0.35 mg/ml solution of Fibroblast Growth Factor 4 (FGF4) protein from 1 mg/ml frozen stock by diluting it with water and store at -20 °C in 30 µl aliquots.
  2. Prepare solutions (either 0.05 or 0.1 mg/ml) of all-trans-retinoic acid (RA) diluted with dimethyl sulfoxide (DMSO) in dark microcentrifuge tubes from a 5 mg/ml stock. Store the stock and 100 µl aliquots at -20 °C.
  3. Prepare solutions (either 2.5 or 5 mg/ml) of BMS 493 (an inverse agonist of RARs) diluted with DMSO in dark microcentrifuge tubes from a 10 mg/ml stock. Store the stock and 100 µl aliquots at -20 °C.
6. Make barriers from aluminum foil. NOTE: The foil used to make the barriers is standard catering foil (measured by micrometer as 0.013 mm thick). Any domestic aluminum foil would likely be suitable.
  1. Cut a 1 cm square piece of aluminum foil and sterilize it by wiping it with 70% ethanol on a tissue. Place it onto a 6 cm sterile plastic Petri dish. Discard the lid and make a lid for the dish from foil. NOTE: This will prevent the cut barriers from later sticking to the plastic lid of the dish due to static.
  2. Place the Petri dish on top of a stage graticule (1 cm long split into 100 sections) under a binocular microscope with magnification set to 1.6X. Using a No.15 blade small scalpel, cut strips of the foil to an exact width, *e.g.*, 0.7 mm or 1.3 mm.
  3. Separate the foil strips using two pairs of No. 4 watchmakers forceps. Then make a right-angled bend in the center of the foil barrier such that it resembles a hinge of the specific width. NOTE: This shape of barrier is taken from ^9, 10^. A hinged barrier stays in place better than a straight, flat barrier. No. 4 forceps are relatively blunt and are used because they are less likely to make dents or scores in the foil barriers.
  4. Line up the barriers in the center of the Petri dish with one side flat against the dish and the other pointing up out of the dish, moving the remaining uncut foil to one side. Prepare 20 or more barriers before operation day. Place the foil lid over the dish and store the barriers in a place where they will not be disturbed. NOTE: Even a slight knock can tip the barriers over or mean that they stick with static to the sides of the dish.
7. Incubate fertilized chicken eggs on their sides on cardboard egg trays at 38 °C for the appropriate length of time before operation day such that the embryos will be at the desired ^8^ stage.

### 2. Preparation of Chick Embryos and Beads on Operation Day

1. 'Window' incubated chicken eggs. NOTE: The following method for opening eggs was passed on by Dennis Summerbell (not published).
  1. With the egg lying on its side, pierce the blunt end (air sac end) of the egg with type AA strong forceps making sure that the inner shell membrane has been broken. To do this grasp the forceps firmly together and use a controlled stabbing motion while holding the egg with the free hand.
  2. Take the egg, still lying on its side, in both hands (one either side) turn it through 180° along the horizontal axis and replace it in the egg tray such that the side that was uppermost is now next to the tray. NOTE: This loosens the embryo from the shell membranes and will mean that the embryo is less likely to be cut when the shell above the embryo is removed.
  3. Take a piece of clear tape approximately 5 cm square and stick this closely over the uppermost surface of the egg to stop the eggshell from crumbling onto the embryo when the egg is opened.
  4. Use the curved scissors to gently break through the shell and its membranes in the center of the uppermost part of the egg, make a very small hole with one blade of the scissors so that air can enter the egg over the embryo (which lies just under the uppermost part of the shell). NOTE: By just breaking the shell membranes and not breaking any of the membranes covering the embryo, air entering the egg will separate the embryo from the shell and the embryo will come to rest on top of the yolk about 0.5-1 cm from the shell.
  5. Use the curved scissors to enlarge the hole in the shell to a circle with a diameter of approximately 1 cm. Remove the piece of excised shell and cover the hole in the egg with a piece of clear tape approximately 5 cm square. To easily remove this tape, press the tape to stick it only at the lip of the hole, leaving the remaining tape free. The free tape can then be grasped to unseal the egg.
2. Stage embryos
  1. Following incubation at 38 °C (see step 1.7) and windowing (step 2.1), use a binocular microscope to stage the chick embryos according to ^8^. Do this just before the eggs are sealed following windowing. Write the stage of each embryo on the eggshell. Return the eggs to the incubator. NOTE: Embryos at stages between 8 and 15 are suitable for barrier and bead experiments. Embryos are more likely to survive if they have not been left out of the incubator for too long prior to operation.
3. Soaking beads prior to operation.
  1. FGF4
    1. Take the number of affinity chromatography gel beads (150-300 µm) that stick to the end of a 1,000 µl pipette tip and wash them in 1 ml phosphate buffered saline (PBS) in a microcentrifuge tube to remove the azide they are stored in. Centrifuge for 5 sec, discard the PBS and repeat the process twice.
    2. Soak the gel beads in a 30 µl drop of 0.35 mg/ml FGF4 protein on a sterile Petri dish on ice for 30 min. Pick beads approximately 150 µm in diameter with No. 5 microforceps for insertion into the embryo. NOTE: Handle ion exchange resin beads used in the following steps with No. 5 microforceps and grip them very gently because the beads are brittle and can shatter if squeezed too tightly. This warning applies to 2.3.2-2.3.3 and all resin bead operations.
  2. RA
    1. Defrost an aliquot of 0.05 or 0.1 mg/ml all-trans-RA diluted with DMSO and place a 100 µl drop on a sterile Petri dish (9 cm diameter), which has previously been covered in foil to protect the RA from light. Take a 10 µl pipette tip and pick up some chloride form ion exchange resin beads on its end and soak these in the RA drop protected from light at room temperature for 20 min.
    2. Rinse the beads in three successive drops of 100 µl Dulbecco's Modified Eagle Medium (DMEM) placed on the same Petri dish at least 1 cm apart. NOTE: The beads take up the phenol red dye from the DMEM. This method is based on ^5^.
    3. Pick beads approximately 100 µm in diameter (beads range in size from 75-180 µm when wet) for insertion into the embryo, using a microscope stage graticule slide under the Petri dish and No.5 microforceps to hold the beads. When picking up the bead from the 100 µl drop of DMEM, first place the bead down onto the Petri dish and remove any remaining DMEM from it either by pushing the bead out of the drop or by placing the closed forceps next to the bead (capillary action draws the liquid away from the bead).
  3. BMS 493
    1. As for the RA (2.3.2), soak ion exchange resin beads in a 100 µl drop of 2.5 or 5.0 mg/ml BMS 493 in DMSO at room temperature for 20 min then rinse in 100 µl drops of DMEM. Pick beads approximately 100 µm in diameter for insertion into the embryo.
  4. DMSO control
    1. As for the RA (2.3.2), soak ion exchange resin beads in a 100 µl drop of DMSO at room temperature for 20 min then rinse in 100 µl drops of DMEM. Pick beads approximately 100 µm in diameter for insertion into the embryo.

### 3. Barrier and Bead Operations


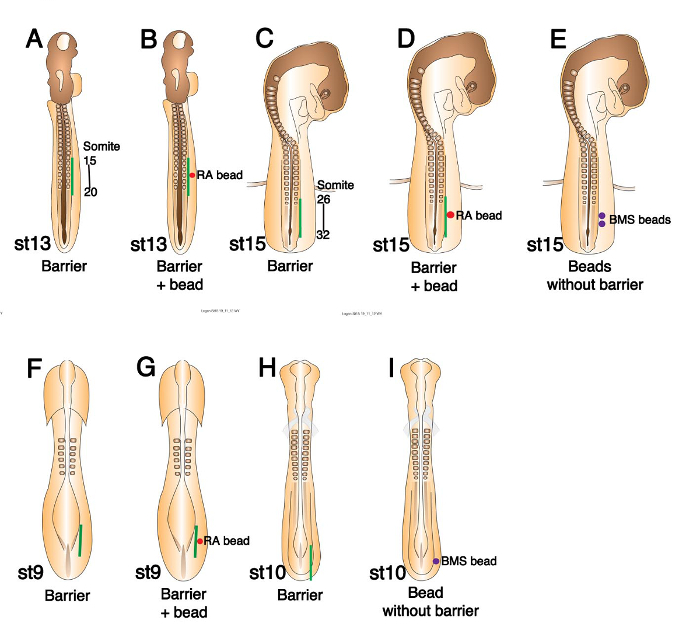
**Figure 1. Schematics of Chick Embryos of Various Stages Showing Where Barriers and or Beads Should be Placed.** (**A**) Stage 13 chick embryo schematic showing barrier position (green line) between somites and LPM at the presumptive wing level (somites 15-20). (**B**) The same schematic as in (A), indicating where RA bead (red circle) should be placed prior to barrier insertion. (**C**) Schematic showing barrier position (green line) at the presumptive leg level (somites 26-32) in a stage 15 embryo. (**D**) The same schematic as in (C), indicating where an RA bead (red circle) should be placed. (**E**) Schematic indicating where BMS 493 beads (purple circles) should be placed in the LPM of a stage 15 embryo. (**F**) Schematic diagram of a stage 9 chick embryo indicating barrier position (green line) between the presomitic mesoderm and LPM at the presumptive wing level. (**G**) Schematic diagram of a stage 9 embryo showing the positions of a barrier (green line) and RA bead (red circle). (**H**) Schematic diagram of a stage 10 chick embryo showing barrier position (green line) at the presumptive leg level. (**I**) Schematic diagram showing BMS 493 bead position (purple circle) in stage 10 leg level LPM. This figure has been modified from ^7^. Please click here to view a larger version of this figure.

1. Barrier inserted at stage 13 to prevent wing bud initiation.
  1. Take a stage 13 embryo from the incubator and place the egg on an egg rest under a binocular microscope set to 3.2X magnification or higher if preferred. Remove the upper layer of clear tape.
    1. Using a steel micro knife, make an incision through the vitelline membrane and lateral plate mesoderm (LPM) adjacent to somites 15 to 20 (**Figure 1A**) on the right side of the embryo (somites have not all formed at this stage). Cut adjacent to the last four somites formed and continue the cut two somite lengths caudally.
    2. Pick up the barrier (0.7-1 mm wide) with No. 5 microforceps at the end protruding from the Petri dish and twist the hand to insert the free end of the barrier into the incision. The hinge shaped barrier lies flat on the vitelline membrane above the LPM with half of it protruding downwards through the incision distal to the somites. As soon as possible seal up the egg with clear tape and return it to the incubator. NOTE: The micro knife and forceps become sticky on contact with the vitelline membrane. It is important to wipe them clean with 70% ethanol before attempting a subsequent operation. This applies to all subsequent operation descriptions and to bead implantations.
    3. If a bead is to be inserted in conjunction with a barrier; firstly, take the bead in No. 5 microforceps and insert it into the cut face of the LPM on the distal side of the incision and then insert the barrier into the incision proximal to the bead (**Figure 1B**). Seal the egg and return it promptly to the incubator.
2. Barrier inserted at stage 15 to prevent leg bud initiation. NOTE: An example of the result of this operation on *Tbox 4 transcription factor* (*Tbx4)* expression is shown in **Figure 2D** and **2E**.
  1. Take a stage 15 embryo from the incubator and place the egg on an egg rest under a binocular microscope set to 3.2X magnification or higher if preferred. Remove the upper layer of clear tape.
    1. Using a steel micro knife make an incision through the vitelline membrane and LPM adjacent to somites 26 to 32 (**Figure 1C**) on the right side of the embryo (somites have not all formed at this stage). Cut adjacent to the last two somites formed and continue the cut five somite lengths caudally.
    2. Pick up the barrier (1.2-1.3 mm wide) with No. 5 microforceps at one end and twist the barrier to insert the free end into the incision. As soon as possible seal up the egg with clear tape and return it to the incubator. NOTE: The hinge shaped barrier lies flat on the vitelline membrane above the LPM with half of it protruding downwards through the incision distal to the somites.
    3. If a bead is to be inserted in conjunction with a barrier, firstly, take the bead in No. 5 microforceps and insert it into the cut face of the LPM on the distal side of the incision and then insert the barrier into the incision proximal to the bead (**Figure 1D**). Seal the egg and return it promptly to the incubator.
    4. If a bead or beads are to be inserted without a barrier, *e.g.*, BMS 493 beads to the leg region at stage 15, do not make the long incision (3.2.2.1). Make a small incision through the vitelline membrane and into the LPM at the position the bead is to be placed. Take the bead in No. 5 microforceps and insert it into the hole in the LPM. Push it in a little further with the closed ends of the forceps (**Figure 1E**).
3. Barrier inserted at stage 9 to prevent wing bud induction and initiation.
  1. Take a stage 8 or 9 embryo from the incubator and place the egg on an egg rest under a binocular microscope set to 3.2X magnification or higher if preferred.
    1. Using a steel micro knife make an incision through the vitelline membrane and LPM lateral and rostral to the primitive streak, at the caudal end of the embryo in line with the somites (**Figure 1F**) about 5 somite lengths long.
    2. Pick up the barrier (0.7-1 mm wide) with No. 5 microforceps at the end protruding from the Petri dish and twist the hand to insert the free end of the barrier into the incision. As soon as possible seal up the egg with clear tape and return it to the incubator. NOTE: The hinge shaped barrier lies flat on the vitelline membrane above the LPM with half of it protruding downwards through the incision.
    3. If a bead is to be inserted in conjunction with a barrier; firstly, take the bead in No. 5 microforceps and insert it into the cut face of the LPM on the distal side of the incision and then insert the barrier into the incision proximal to the bead (**Figure 1G**). Seal the egg and return it promptly to the incubator.
4. Barrier inserted at stage 10 to prevent leg bud induction and initiation. NOTE: An example of the result of this operation on *Tbx4* expression is shown in **Figure 2G**.
  1. Take a stage 10 embryo from the incubator and place the egg on an egg rest under a binocular microscope set to 3.2X magnification or higher if preferred.
    1. Using a steel micro knife make an incision through the vitelline membrane and LPM lateral to the primitive streak, at the caudal end of the embryo in line with the somites (**Figure 1H**) approximately 6 somite lengths long.
    2. Pick up the barrier (1.2 mm wide) with No. 5 microforceps at the end protruding from the Petri dish and twist the hand to insert the free end of the barrier into the incision. As soon as possible seal up the egg with clear tape and return it to the incubator. NOTE: The hinge shaped barrier lies flat on the vitelline membrane above the LPM with half of it protruding downwards through the incision.
    3. If a bead is to be inserted without a barrier, *e.g.,* BMS 493 bead to the leg region at stage 10, make a small incision through the vitelline membrane and into the LPM at the position the bead is to be placed. Take the bead in No. 5 microforceps and insert it into the hole in the LPM. Push it in a little further with the closed ends of the forceps (**Figure 1I**).
5. 24 hr or more after operating check the position of the barrier compared with the emerging contralateral limb bud. Discard any embryos in which the barrier is clearly not opposite the left limb bud. In this case the barrier has been wrongly positioned.

### 4. Harvesting Embryos, Fixation and Preparation for Wholemount *In Situ* Hybridization (WISH)

1. Harvesting embryos post operation.
  1. Harvest embryos at stage 19-23 such that limb bud morphology is clearly visible in both the operated and contralateral control sides.
    1. Using curved scissors cut a larger hole in the eggshell. Cut around the embryo while holding the large blood vessel to the left of the embryo with microforceps. Use this grip of the vessel to remove the embryo and place it in a Petri dish of phosphate buffered saline (PBS) pH 7.3.
    2. Under a binocular microscope, dissect away extra embryonic vessels and membranes using a combination of curved scissors and no. 5 microforceps. Remove the head using the scissors.
2. Fixation of embryos post harvesting.
  1. Using curved blunt forceps, lift the embryo and place it in 5 ml 4% paraformaldehyde (PFA) in PBS at room temperature for two hr rocking and then at 4 °C overnight in a 20 ml glass bottle with a screw cap. Caution: PFA is a serious hazard to health. It is a harmful, corrosive irritant and the powder must be prevented from coming into contact with skin, eyes and lungs. Use personal protective clothing and a fume hood while preparing the solution. Once it is a 4% solution, gloves, eye protection and a lab coat must be worn. NOTE: It is important to get the embryos into fix quickly to best preserve mRNA integrity.
3. Removal of barriers prior to wholemount *in situ* hybridization. NOTE: Barriers are removed because they are sharp and can damage embryos as they go through the *in situ* hybridization protocol, especially if several embryos are stained at once.
  1. Following fixation, place the embryo in a dish of PBS under a binocular microscope.
  2. Using two pairs of No. 5 microforceps, place the left pair closely either side of the barrier in the operated side of the embryo. Grasp the barrier with the right pair and gently pull the barrier from the embryo using the left pair to keep the embryo down and to prevent any tissue from adhering to the barrier as it is removed.
4. Carry out WISH essentially as described in ^11^.

## Representative Results

The protocol detailed above describes the methods employed in Nishimoto *et al.*, 2015. The paper studies limb bud induction and initiation in both the fore and hindlimb of the chick embryo. Results following the insertion of impermeable barriers to prevent the outgrowth of the forelimb bud have been published previously ^1, 2, 9^ but there has been only one study describing barriers preventing leg bud initiation ^1^. Hindlimb data from Nishimoto *et al.*, 2015 are presented here as representative results of the study of mRNA expression following barrier insertion.

Tbx4 Expression in the LPM Is Not Sufficient to Initiate Hindlimb Outgrowth: **Figures 2B **and** C** (**Table 2**) show that barrier insertion at stage 15 (**Figure 2A**) (see protocol 3.2) leads to both *Fgf10* and *Fgf8* expression being absent in the leg bud region (denoted by bracket) on the operated right side. Compare with the unoperated left side. However, *Tbx4* expression is observed at both stage 19 and stage 23 on the operated side (**Figure 2D **and** E**). We conclude that *Tbx4* expression alone is not sufficient to initiate hindlimb outgrowth from the LPM. Other studies in the chick have suggested that *Tbx* gene expression is sufficient to initiate forelimb bud formation ^12, 13^.

**Figure 2C'** is included to illustrate how a barrier looks imbedded in the leg region of the embryo post fixation. In the majority of cases barriers were removed prior to *in situ* hybridization (protocol 4.3).

RA from the Somites Is Essential in the LPM before *Fgf10* Induces Limb Bud Outgrowth: **Figure 3A** is a schematic representing the addition of an RA soaked bead distal to a barrier inserted at stage 15 (protocol 3.2.1.3). Subsequent to this operation limb bud outgrowth (denoted by an asterisk) is observed on the operated right side and *Tbx4*, *Fgf10* and *Fgf8* are expressed in the bud as they are in the control left side (**Figure 3B**,** C **and** D**,** Table 2**). Thus addition of RA to the LPM overcomes the effect of the barrier and limb bud initiation proceeds. We conclude that in normal development RA from the somites is essential in the LPM before limb bud outgrowth can be initiated. The resulting hindlimb buds are generally smaller than those on the control side. This may be due to the RA dose in the LPM being not equivalent to the wild type situation. If the RA dose is too high the Apical Ectodermal Ridge (AER) can be shortened and smaller buds would result ^14, 15^.

To confirm that RA (from the somites) in the LPM is required for normal limb bud initiation an inverse agonist of RAR, BMS 493, was used. Beads are implanted into the leg region LPM at stage 15 (see protocol 3.2.1.4) (**Figure 3E**). *Fgf10* expression is downregulated following application of BMS 493 beads in the LPM, resulting in smaller hindlimb buds compared to the control side (**Figure 3F**; **Table 3**). Control DMSO beads do not cause these defects (**Figures 3G **and** 3H**; **Table 3**). The defects observed following BMS 493 application are milder than those induced by a barrier operation; *i.e.,* *Fgf10* is still expressed and limb buds are formed. This is likely to be because the effects of BMS 493 are restricted locally around the beads and may not be able to antagonize all the RA produced by axial tissues.

Early Axial Signals Specify the LPM Cells that Later Express *Tbx4:* We tested when the LPM acquires its ability to express *Tbx4 *in the prospective leg-forming region. The earliest stage at which a barrier can be inserted that will subsequently block leg bud outgrowth, is stage 10. We are the first to develop a protocol to insert barriers at stages earlier than stage 12. Barriers inserted between the paraxial mesoderm and the presumptive leg LPM at stage 10 (see protocol 3.4) block leg bud formation and the expression of *Tbx4* in the leg-forming LPM (**Figures 2F** and **2G**, **Table 2**), suggesting that a signal from axial tissues at stage 10 is required for later expression of *Tbx4* in hindlimb LPM. Compare this result with **Figures 2D **and** E** where later barrier insertion allows *Tbx4* expression to be established. Furthermore, we tested whether this axial signal could be RA by observing whether the inverse agonist of RAR reduces *Tbx4* expression. A BMS 493 bead placed in the hindlimb LPM at stage 10 (see protocol 3.4.1.3) downregulates *Tbx4* expression (**Figures 2H **and** 2I**), whereas control beads soaked in DMSO do not affect *Tbx4 *expression (**Figures 2J **and** 2K**). Together, these results support a model that an RA signal from axial tissues regulates limb induction by positively regulating *Tbx4* in the hindlimb LPM ^7^.

Post-operative Death: **Tables 1, 2 **and** 3** give details of experimental outcomes including the number of chick embryos that died post operation and before harvesting. Some deaths are to be expected after microsurgery of this nature. The numbers dying can be kept to a minimum by making sure that instruments are clean, the hole in the egg is the minimum size required, that the embryos are left without the protection of the sticky tape seal for a minimum time and, in conjunction with this last point that the operation is carried out as quickly as possible. None of these operations are very time consuming but operations involving beads and barriers take a little more time. Despite these measures, operations carried out at the earliest stages and those in the wing region are more likely to result in death. There are large blood vessels around the limb-forming regions and accidental rupture of these vessels may lead to death due to blood loss. We find that eggs opened at stage 8 or 9, whose embryos have not been operated on, will often die by the next day. Therefore, the only solution to these problems is to carry out a high number of experiments.


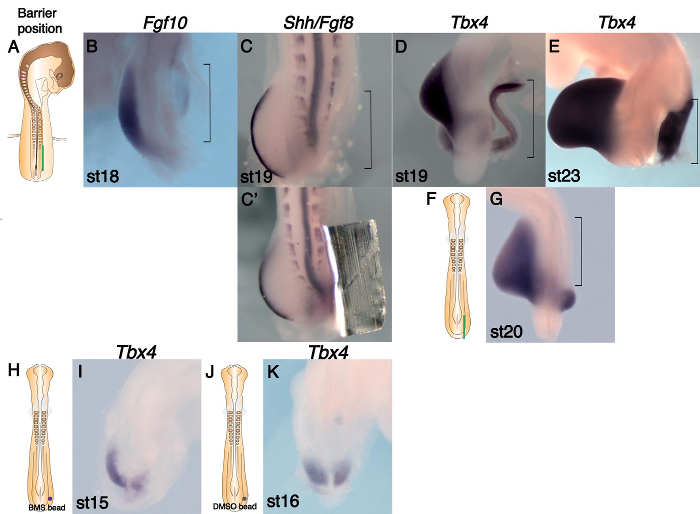
**Figure 2.Marker Gene Expression Changes following Barrier or Bead Insertion at Presumptive Leg Level. **(**A**) Schematic showing barrier position (green line) at the presumptive leg level (somites 26-32) in a stage 15 embryo. (**B**-**E** and **G**) WISH analysis on operated embryos. The leg region is outlined (brackets). (**B**) Fgf10 expression is absent in the operated LPM, but robust expression is detected in the left bud. (**C**) *Fgf8* expression is absent in the operated side, suggesting there is no AER. (**C'**) The same embryo before the barrier was removed. (**D**) Tbx4 is expressed in the LPM at the same rostro-caudal level as the control bud. (**E**) Tbx4 expression is maintained in the right leg region despite absence of limb growth. (**F**) Schematic diagram of a stage 10 chick embryo showing barrier position (green line) at the presumptive leg level. (**G**) Tbx4 expression is absent in the operated right LPM (bracket). (**H**) Schematic diagram of a stage 10 chick embryo showing BMS 493 bead position (purple circle) at the presumptive leg level. (**I**) Tbx4 expression is downregulated on the operated right side following treatment with BMS 493. (**J**) Schematic diagram of a stage 10 chick embryo showing control DMSO bead position (gray circle). (**K**) Tbx4 is expressed on the operated right side at a similar level to that of control left side following treatment with DMSO alone. This figure has been modified from ^7^. Please click here to view a larger version of this figure.


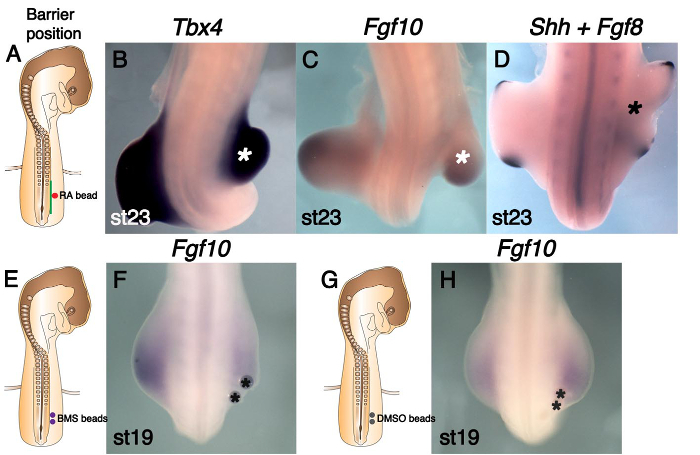
**Figure 3. RA Rescues Limb Bud's Absence Caused by Barrier Insertion and is Essential in the LPM Prior to Leg Bud Outgrowth.** (**A**) Schematic diagram indicating barrier position (green line) at the presumptive leg level (somites 26-32) and an RA-soaked bead (red circle). (**B**-**D**) RA rescues leg bud outgrowth (shown by an asterisk). (**B**) Tbx4 expression is present in the rescued leg bud similar to the un-operated side. (**C**) *Fgf10* is expressed in the rescued leg bud similar to the un-operated side. (**D**) *Fgf8* is expressed in the AER of the rescued right leg bud. (**E**) Schematic indicating where BMS 493 beads (purple circles) were placed in the presumptive leg LPM of stage 15 embryos. (**F**) *Fgf10* expression is downregulated on the operated right side and the leg bud is small following BMS 493 treatment. Beads are asterisked. (**G**) Similar schematic to (E), indicating the position of control DMSO beads (gray circles). (**H**) DMSO beads (shown by asterisks) did not affect *Fgf10* expression or leg bud size. This figure has been modified from ^7^. Please click here to view a larger version of this figure.

**Table:** 

Stage barrier placed (+ bead) *	**Number operated**	Wing missing **	Wing present **	**Number dead**
Stage 12-14	40	24	1	15
Stage 8/9	65	23	0	42
Stage 12-13 (FGF4 bead)	41	7 (bead missing)	17	17
Stage 12-13 (RA bead)	75	9 (bead missing)	32	34
Stage 13 (control bead)	5	3	0	2
* 0.7-1 mm foil barriers placed between somites 15-20 and the lateral plate mesoderm. 0.35 mg/ml FGF4 bead. 0.05-0.1 mg/ml RA bead.
DMSO control bead.
** Chick embryos fixed stages 21-23

**Table 1. Wing Level Barrier and Bead Experiment Outcomes.**

**Table:** 

Stage barrier placed (+ bead) *	**Number operated**	Leg missing **	Leg present **	**Number dead**
Stage 12-15	43	39	0	4
Stage 10/11	23	16	0	7
Stage 15 (FGF4 bead)	8	0	5	3
Stage 15 (RA bead)	18	0	18	0
* 0.7-1.3 mm foil barriers placed between somites 26-32 and the lateral plate mesoderm. 0.35 mg/ml FGF4 bead. 0.05-0.1 mg/ml RA bead.
** Chick embryos fixed stages 18-24

**Table 2. Leg Level Barrier and Bead Experiment Outcomes.**

**Table:** 

**Number operated**	**Leg bud small**	**Leg bud slightly small**	**Leg bud normal size**	**Number dead**
BMS 493 beads *				
15	12	3	0	0
DMSO beads **				
12	0	3	9	0
* 2-3 beads soaked in 5 mg/ml BMS 493 placed in the right stage 14-15 LPM at leg level.
** 2-3 beads soaked in DMSO alone placed in the right stage 15 LPM at leg level.
Chick embryos fixed at stages 17-19.

**Table 3. Leg Level BMS 493 Bead Experiment Outcomes.**

## Discussion

We describe the use of impermeable barriers to prevent limb bud formation in the chick embryo. This technique has a number of critical steps. Following preliminary experiments, we found that the hinge shaped barriers used in ^9, 10^ remain in place in the embryo far better than straight barriers. The distal flat side of the barrier sticks to the vitelline membrane and keeps the barrier in place (**Figure 2C'**). Careful preparation of hinge shaped barriers from aluminum foil as described in protocol step 1.6 is essential to the success of the operation. A second important step is to ensure that eggs are not left out of the incubator for too long following windowing and staging prior to operating on the embryo. In our experience, embryos survive an operation better if they are at or near incubation temperature when the operation is carried out. Operations should therefore be carried out as quickly as possible and the eggs resealed promptly to ensure good survival rates. Many researchers add a drop of antibiotics to the embryo following microsurgery before returning the eggs to the incubator. The presence of the liquid prevented the barriers from sticking well in position. Addition of antibiotic could also dislodge beads after placement. Therefore not using antibiotics, swift operations and good cleanliness with instruments is recommended to avoid infection. Wiping instruments with 70% ethanol following every stage of an operation is essential to prevent instruments from becoming sticky from contact with the vitelline membrane which can cause both barriers or beads to adhere to forceps (see protocol step 3.1.2.2). Both varieties of beads used could fall out of place following the operation. When the barrier position was checked the next day it was not possible to see through the barrier to ascertain whether the bead remained in place or not. In cases where the bead had fallen out wing bud outgrowth was not rescued (**Table 1**). Avoiding transferring liquid with the bead when implanting them means that beads stick more easily to the cut face of the LPM (see protocol 2.3.2.3). Finally ensuring that embryos are fixed immediately after harvesting is critical to good subsequent gene expression analysis (see protocol step 4.2).

This protocol modifies previously published experiments that used barriers to prevent limb bud initiation, defines specific optimum widths for barriers and introduces combining barrier and bead placement followed by gene expression analysis. We found using conventional aluminum foil, which is cheap and readily available in all labs, produces outcomes equal to those that previously used tantalum foil. The foil can be left in the embryo when carrying out *in situ* hybridization (**Figure 2C'**) but we usually remove it if more than one embryo is being processed to prevent the sharp edges of the foil damaging embryos. Pilot studies demonstrated that the width of leg region barriers is particularly important. Initially the barriers trialed were too short and leg bud outgrowth was not prevented. 1.2-1.3 mm is the optimum width for a barrier to block leg bud outgrowth completely. The narrowest wing barriers that one can place accurately to prevent bud outgrowth give the best survival rates. This can be as narrow as 0.5-0.7 mm. However, barriers that are a little wider are more likely to result in complete blocking of wing bud outgrowth because they allow for a little inaccuracy of placement (we used 0.7-1 mm). Avoidance of blood vessels when making the incision is critical for wing bud level barriers. Semi-permeable barriers have previously been used to bisect the chick limb bud. MacCabe and Parker, 1976 and Summerbell, 1979 both used filters 0.45 µm and 0.8 µm pore size, respectively ^16, 17.^ They reported that diffusible signals could pass through the pores in the filters and that results gained using semi-permeable barriers were midway between a limb bud with no barrier present and one with an impermeable tantalum foil barrier. This protocol could be adapted to use semi-permeable barriers of varying pore size to differentiate candidate signals based on size or to modify the dynamics of signal diffusion.

This protocol has various limitations mainly associated with the model organism itself. Such techniques could be used to probe further questions in limb development and also other questions of vertebrate embryogenesis. A proviso is that the organ/area in question should be developing when the chick embryo is accessible to intervention. The presence of large blood vessels in the area where a barrier needs to be placed makes barrier placement later than stage 13 or 14 technically challenging. If large blood vessels are cut during barrier placement this can cause embryo mortality. Our investigation of when the transcription factors *Tbx5* and *Tbx4* are first induced in the LPM was limited by the earliest stage at which we could place a barrier that would later end up opposite either the wing or leg bud respectively. It could be illuminating to attempt to block *Tbx *gene induction at earlier stages, closer to gastrulation. The earliest a barrier could be placed in the presumptive wing region successfully was stage 8 and in the leg region stage 10. This protocol is probably not suitable for the study of gene expression beyond limb bud stages because subsequent rapid growth would displace the barrier and results would relate only to the initial position of the barrier.

Having related the limitations of the chick embryo in this context, its advantages over other vertebrate model organisms are many. The chick embryo is a very suitable animal model for this type of physical intervention in a vertebrate embryo. The embryos are relatively large and are easily accessible through the eggshell. One can return to the embryo for further experimentation (*e.g.,* the removal of a bead ^18^ or even a barrier) or to check on progress by simply removing the tape window. There is possible scope to include live imaging or time lapse imaging. We describe a method of windowing chicken eggs that is particularly suited to operating on early stage embryos. In the case of limb studies it is very important and helpful that there is a contralateral control limb for every experiment that serves as an ideal internal control for each biological replicate.

The combination of using impermeable barriers to prevent limb outgrowth and beads soaked in signaling molecules has not been reported before. Our analysis of mRNA expression following such interventions is also novel. These techniques enabled us to dissect the difficult problem of when limb specific transcription factors *Tbx5* and *Tbx4* are induced in the LPM and also to show that later, prior to limb bud initiation, the presence of these *Tbx* genes in the LPM is not sufficient for the activation of *Fgf10* transcription and the start of limb bud outgrowth. **Figures 2G, 2D **and** 2E** illustrate these findings well. The absence of *Tbx4* expression following the insertion of a barrier at stage 10 in **Figure 2G** is in stark contrast to its presence in 2D and 2E following barrier insertion at stage 15, after the induction of *Tbx4*. The implantation of RA soaked beads facilitated the rescue of limb bud initiation following barrier insertion and so points to RA from the somites being essential in the LPM before limb bud outgrowth can begin. Such techniques could be used to solve further questions in limb development but also other questions of vertebrate embryogenesis. Areas of the brain, eye and trunk are likely to be suitable.

## Disclosures

The authors declare they have no competing financial interests.
